# The relationship of dyspnea and disease severity with anthropometric indicators of malnutrition among patients with chronic obstructive pulmonary disease

**DOI:** 10.12669/pjms.346.15769

**Published:** 2018

**Authors:** Mirza Muhammad Ayub Baig, Naheed Hashmat, Muhammad Adnan, Tayyaba Rahat

**Affiliations:** 1 *Dr. Mirza Muhammad Ayub Baig, FCPS. Assistant Professor of Pulmonology, Department of Pulmonology, Sir Ganga Ram Hospital, Lahore, Pakistan*; 2 *Dr. Naheed Hashmat, FCPS. Associate Professor of Medicine, Department of Medicine, Sir Ganga Ram Hospital, Lahore, Pakistan*; 3 *Mr. Muhammad Adnan, M.Sc., Research Officer, PHRC Research Center, Fatima Jinnah Medical University, Lahore, Pakistan*; 4 *Ms. Tayyaba Rahat, M.Phil., Statistical Officer, PHRC Research Center, Fatima Jinnah Medical University, Lahore, Pakistan*

**Keywords:** Body mass index, Chronic obstructive pulmonary disease, Dyspnea, Malnutrition

## Abstract

**Objective:**

To find the association of dyspnea and disease severity with anthropometric indicators of malnutrition among chronic obstructive pulmonary disease patients.

**Methods:**

The cross-sectional analytical study was carried out at Sir Ganga Ram Hospital, Lahore during October 2013 to December 2014. Total 138 adult patients with severe COPD were enrolled. The severity of disease was measured by global initiative for chronic obstructive lung disease criteria; and dyspnea was assessed by modified medical research council dyspnea scale. Anthropometric indicators of malnutrition such as body mass index (BMI) and mid upper arm circumference (MUAC) were measured to evaluate the nutritional status of COPD patients. Data was analyzed by using Statistical Package for Social Sciences version 20.

**Results:**

The mean age of 138 patients was 55±3 years. The frequency of male patients (76.8%) was three-times higher than female patients (23.2%). The overall frequency of underweight patients measured by BMI was 44%, which was increased to 92% undernourished patients by using MUAC. When compared with female patients, the male patients showed lower means of BMI, MUAC, FEV_1_% and FEV_1_/FVC ratio. The significant relationship of high grade dyspnea with BMI (p=0.001), and MUAC (p=<0.001) revealed that malnourished COPD patients had more shortness of breathing as compared to normal-weight patients. Similarly, the association of FEV_1_% with BMI (p=0.001), and MUAC (p=<0.001) showed that malnourished patients had very severe type of COPD than normal-weight patients.

**Conclusion:**

Dyspnea and severity of disease had significant association with BMI and MUAC among COPD patients. Thus, assessment of nutritional status by measuring BMI and MUAC should be considered to predict the severity of disease among adult COPD patients.

## INTRODUCTION

Chronic obstructive pulmonary disease (COPD) is a common respiratory condition characterized by airflow limitation.^1^ The World Health Organization (WHO) reported that global prevalence of COPD in 2016 was 251 million cases; mortality rate in 2015 was 3.17 million deaths; and >90% of deaths occurred in low and middle income countries.^2^ In Pakistan, the prevalence of COPD in general population of Karachi was 13.8%.^3^ There are four stages of COPD ranging from stage I to IV; and severity of disease increases with increase in the stage number which is usually determined by FEV1% level.^4^

Active as well as passive exposure to tobacco smoking, air pollution, infectious diseases and genetic disorders are the main causes of COPD.^5^ The common symptoms of COPD include dyspnea, cough, chest pain, and wheezing; while age, nutritional status, and physical disability are the factors that affect dyspnea.^6^ The screening and treatment of COPD patients for malnutrition can improve their nutritional status, disease severity and outcome.^7^,^8^ Weight loss is an important negative prognostic factor and its management can improve the prognosis in COPD patients.^9^ According to the National Institute for Health and Clinical Excellence (NICE) guidelines, calculation of body mass index (BMI) is recommended in all COPD patients.^10^ However, measuring mid upper arm circumference (MUAC) is a simple and effective tool than BMI.^11^ The outcome of malnutrition and COPD is also associated with increased healthcare costs.^12^ Therefore aim of the study was to find the association of dyspnea and disease severity with BMI and MUAC in COPD patients.

## METHODS

The cross-sectional analytical study was carried out from October 2013 to December 2014 at Pulmonology Clinic of Sir Ganga Ram Hospital, Lahore. The study was approved by Institutional Review Board/ Ethics Review Committee, Fatima Jinnah Medical University, Lahore via letter No.19-Art/Pulmon-IRB/FJ. Informed written consent was obtained from all patients. Total 138 patients with severe COPD were enrolled by non-probability purposive sampling technique. Global initiative for chronic obstructive lung disease (GOLD) criteria was used to measure the severity of COPD.^13^ Height (cm) and weight (Kg)were measured to calculate BMI.^10^ MUAC was measured at the left arm at midpoint between tip of shoulder and tip of elbow.^14^ Perception of dyspnea was assessed by modified medical research council(MMRC) dyspnea scale.^15^ Data was analyzed by using Statistical Package for Social Sciences (SPSS) version 20.

## RESULTS

The mean age of 138 adult patients with severe COPD was 55±3 years. The frequency of male patients (76.8%) was three-times higher than female patients (23.2%). Other characteristics of study population included 20.3% illiterate; 82.6% current smokers and 14.5% ex-smokers. Patients categorized to severe and very severe COPD were 15% and 85%, respectively. Dyspnea grade<4 was present in 12% patients; and Dyspnea grade ≥4 in 88% patients.

The mean BMI of overall population was within normal limits but more prone towards underweight cutoff. When compared with female patients, the male patients showed lower means of BMI, MUAC, FEV_1_% and FEV_1_/FVC ratio. It was obvious from the results that means of undernutrition indicators and disease severity among males were poor (Table-I).

**Table-I:** Comparison of anthropometric measures and lung function tests.

Variables	Male (n=106)	Female (n=32)	Total (n=138)
Height (cm)	164±9	158±9	162±9
Weight (Kg)	50±5	49±5	50±5
BMI (Kg/m^2^)	18.7±2.0	19.6±2.2	18.9±2.1
MUAC (cm)	19±2	20±2	19±2
FEV_1_ (% predicted)	27±4	29±6	28±5
FEV_1_/ FVC ratio	0.34±0.5	0.44±0.5	0.36±0.4

BMI: Body mass index, MUAC: Mid-upper arm circumference, Forced expiratory volume in 1 second, FVC: Forced vital capacity.

The overall frequency of underweight patients measured by BMI was 44%, which was increased to 92% undernourished patients by using MUAC. However, frequency of malnourished males remained higher than females by using either BMI or MUAC (Table-II).

**Table-II:** Gender wise frequency of normal and malnourished patients.

		Male (n=106)	Female (n=32)	Total (n=138)
BMI	Underweight	50 (47%)	11 (34%)	61 (44%)
	Normal	56 (53%)	21 (66%)	77 (56%)
MUAC	Undernourished	100 (94%)	27 (84%)	127 (92%)
	Normal	06 (06%)	05 (16%)	11 (08%)

Column %

The significant relationship of high grade dyspnea with BMI (p=0.001), and MUAC (p=<0.001) revealed that malnourished COPD patients had more shortness of breathing as compared to normal-weight patients (Table-III).

**Table-III:** Association of dyspnea with anthropometric indicators of malnutrition.

		Dyspnea			P-value

		Grade ≥4	Grade <4	Total	
BMI	Underweight	60	01	61	0.001
	Normal	61	16	77	
	Total	121	17	138	
MUAC	Undernutrition	118	09	127	<0.001
	Normal	03	08	11	
	Total	121	17	138	

BMI: Body mass index; MUAC: Mid upper arm circumference.

Similarly, the association of FEV_1_% with BMI (p=0.001), and MUAC (p=<0.001) showed that malnourished patients had very severe type of COPD than normal-weight patients. The significant association was also present between high grade dyspnea and FEV_1_% (p=<0.001)(Table-IV). These findings evidenced that high grade dyspnea and disease severity had statistically significant associations with anthropometric measures among adult severe COPD patients.

**Table-IV:** Association of disease severity with dyspnea and anthropometric measures.

		FEV_1_ % predicted			P-value

		<30%(Very Severe)	30-49%(Severe)	Total	
BMI	Underweight	59	02	61	0.001
	Normal	58	19	77	
	Total	117	21	138	
MUAC	Undernutrition	113	14	127	<0.001
	Normal	04	07	11	
	Total	117	21	138	
Dyspnea	Grade ≥4	113	08	121	<0.001
	Grade <4	04	13	17	
	Total	117	21	138	

FEV: Forced expiratory volume; BMI: Body mass index; MUAC: Mid upper arm circumference.

## DISCUSSION

Dyspnea or shortness of breath is the most common symptom of COPD which is affected by different factors such as age, nutritional status, and physical disability.^6^ Malnutrition is associated with weight loss in COPD patients, thus its management can improve the prognosis of COPD.^9^ Nutritional supplementation to severely ill COPD patients can play an important role.^16^ Based on the knowledge described above; the present study was aimed to determine the relationship of dyspnea and disease severity with anthropometric indicators of malnutrition among COPD patients.

It was revealed in the present study that the majority of COPD patients were elderly; males were more affected than females; and frequency of cigarette smokers was very high. Similar findings for elderly and heavy smoking but no significant gender differences were reported by Prescott et al.^17^

The calculation of BMI in all COPD patients is recommended by the NICE guidelines.^10^ Unfortunately, a very high number of underweight COPD patients were found in the study. Moreover, a significant association of dyspnea and disease severity with low BMI was determined. Almost similar frequency of underweight COPD patients and an association of FEV1% with BMI have been reported by Ardestani et al.^18^ Mitra et al. reported that age had a direct relationship with the severity of disease; whereas BMI had an inverse association with disease severity.^19^ These relationships showed that either increase in the age of COPD patients or decrease in BMI may increase the severity of disease. But Ischaki et al. found no association between BMI and different stages of COPD. However, concluded that Fat-free mass index (FFMI) was more accurate than BMI in expressing severity of disease.^20^

Interestingly, the frequency of undernutrition by using MUAC reached to 92%, which was more than double of underweight assessment by BMI. However, number of affected males remained higher than of females. Likewise BMI, similar significant association between MUAC and severity of disease was obtained in current study. Slightly differing from these findings, Ardestani et al. reported that MUAC had more significant association with disease severity than BMI.^18^

The results of present study have validated the findings from previous studies that anthropometric measurements such as BMI and MUAC have statistically significant association with high grade dyspnea and disease severity. It is also well established that the screening and treatment of COPD patients for malnutrition can improve their nutritional status, disease severity and outcome.^7^,^8^ So, nutritional therapies for muscle strengthening along with pharmacological therapy must be focused to relieve dyspnea.^16^

## CONCLUSION

Dyspnea and severity of disease had significant association with BMI and MUAC. Therefore, it is suggested that assessment of MUAC and BMI should be considered as prognostic marker of disease severity among COPD patients.

### Author`s Contribution

**MMAB** conceptualized, did data collection, review and final approval of manuscript.

**NH** did data collection & editing of manuscript.

**MA** did data analysis, data interpretation, & manuscript writing.

**TR** did statistical analysis & editing of manuscript.

All authors approved the final version of the manuscript.
